# Improving social emotional functioning in adolescents with Developmental Language Disorders: A mini review and recommendations

**DOI:** 10.3389/fpsyt.2022.966008

**Published:** 2022-12-09

**Authors:** Elke Arts, Bram Orobio de Castro, Ellen Luteijn, Ben Elsendoorn, Constance T. W. M. Vissers

**Affiliations:** ^1^Behavioural Science Institute, Radboud University, Nijmegen, Netherlands; ^2^Royal Kentalis, Utrecht, Netherlands; ^3^Research Institute of Child Development and Education, University of Amsterdam, Amsterdam, Netherlands; ^4^Royal Kentalis, Secondary School for Special Education for Children and Adolescents With Language and Communication Problems, Arnhem, Netherlands

**Keywords:** social emotional functioning, language, social dialogue, self-directed speech, Executive Functioning, Theory of Mind, Developmental Language Disorder, Virtual Reality

## Abstract

Adolescents with Developmental Language Disorders (DLD) have more difficulties in social emotional functioning than their typically developing peers (TD), such as shyness and anxiety in social situations, fewer peer relations, greater risk of victimization, social isolation and depression. In addition, they are more likely to report higher levels of hyperactivity and conduct problems. These problems derive from a complex interplay between difficulties in language, social communication, underlying cognitive deficits in Theory of Mind (ToM), Executive Functioning (EF) and self-directed speech (SDS). The aim of this mini review is to provide an overview of studies examining the effectiveness of interventions targeting the factors underlying social emotional functioning of school-aged children and adolescents with DLD. We found that studies dedicated to social emotional functioning in school-aged children and adolescents with DLD were relatively scarce. Based on this overview, we give suggestions to improve social emotional functioning in adolescents with DLD. We propose that intervention programs should target the social, linguistic and cognitive functions underlying social emotional functioning and create opportunities to practice these skills in daily, real-life situations with peers.

## Introduction

Adolescents with Developmental Language Disorders (hereafter: DLD) have problems in their receptive and/or expressive language skills, in the absence of any biomedical etiology (1). Expressive language refers to the ability to produce spoken sounds and comprehensible language. Receptive language refers to the ability to understand and comprehend the language that you hear or read. While linguistic deficits are generally seen as the primary problem in DLD, hypotheses that concentrate solely on language fail to explain the heterogeneous problems adolescents with DLD may experience (2). One of these problems is that adolescents with DLD tend to demonstrate more difficulties in social-emotional functioning than their typically developing (hereafter: TD) peers (3–6). While these difficulties already occur in preschool children with DLD, they seem to increase into adolescence (7).

The increased problems in social emotional functioning in adolescents with DLD underscore the need for an intervention method to improve their social-emotional functioning. However, to the best of our knowledge, there is no adequate program for improving social-emotional functioning in adolescents with DLD at present. Developing effective support for youth with DLD requires a thorough understanding of factors underlying the social-emotional problems. To this end, we will first describe a theoretical framework of factors that may contribute to social emotional problems in DLD and provide suggestions for tailored intervention. Second, we provide an overview about existing studies testing the effectiveness of interventions targeting skills underlying social emotional functioning. Finally, we suggest potential ways to improve social emotional functioning in adolescents with DLD.

## The interplay between language, social communication, and cognitive functions in social emotional functioning

Social emotional problems of adolescents with DLD can be explained from a neurocognitive perspective. More specifically, children with DLD have deficits in Executive Functioning (EF) (8–10) and Theory of Mind (ToM) (11–14). EF can be defined as higher cognitive control processes, to regulate human cognition and behavior, in order to achieve goals (15, 16). ToM refers to the ability to understand emotions (affective), thoughts, intentions, desires and beliefs (cognitive) of oneself (intrapersonal) and others (interpersonal) (17).

In turn, EF and TOM have been proposed as predictors of social emotional functioning (5, 18, 19). Adequate social-emotional functioning requires children to understand and predict behavior and represent mental states of oneself and others (i.e., ToM) (5). In addition, adequate social-emotional functioning depends on well-developed inhibition, working memory and cognitive flexibility (i.e., the main concepts of EF) (20, 21). It is expected that children will inhibit their unnecessary thoughts and inappropriate behavior (inhibition), demonstrate flexibility in unique social situations (cognitive flexibility) and store their social information (working memory).

There are two reasons to suggest that the development of EF and ToM is completely intertwined with the development of language. The first reason is that children with DLD, from early childhood on, have impairments in language as well as in EF and ToM (22, 23). The second reason is that EF and ToM are proposed to develop in a hierarchical matter. During the first years, children will develop relatively simple EF (e.g., behavioral attention) and ToM (e.g., imitation) components. These relatively simple functions form the basis of the development to more complex EF (e.g., planning) and ToM (e.g., false belief) functions later in life. Since language develops in a comparative manner, the early development of language, EF and ToM plausibly interact in an empowering or inhibitive manner. In this way, recent authors have argued that the deficits in EF and ToM in individuals with DLD can be explained by the problems in the synthetization of language with precursors of EF and ToM (22–24).

There is considerable evidence that cognitive deficits in DLD not only appear from difficulties with social dialogue, but also from difficulties with the inner dialogue (25). In words of (26), social communication transforms into an internalized “conversation” with the self (i.e., self-directed speech) *via* internalization. Within this development through internalization, children will acquire the ability to use language to regulate their behavior (i.e., EF). In addition, this process of internalization through social dialog implies that different perspectives and beliefs expressed by others are also internalized through language. This process will provide the basis for the ability to further adopt perspectives of others, and thus the development of ToM (27). However, this process of internalization appears to be delayed in children with DLD (28, 29).

According to transactional models of developmental psychopathology, the effects of adolescents' neuropsychological and language development on their social emotional functioning are likely bidirectional: Less developed social-emotional skills are likely to evoke less rich social interactions with peers and adults, which will in turn limit the social learning experiences of adolescents with DLD. Thus, social problems of adolescents with DLD may compromise the development of EF, TOM, and (social) language.

**Figure 1:**
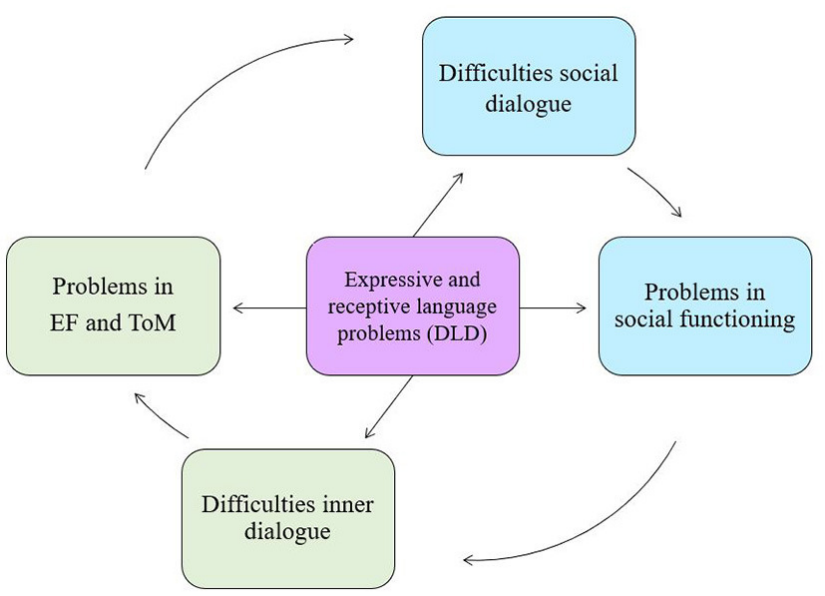
Theoretical model of problems in social functioning, EF and ToM in DLD. Adapted from Vissers et al. (30), p.84.

In summary, social emotional functioning by adolescents with DLD seems to be hampered by a vicious cycle of deficits in social communication, self-directed speech and cognitive skills (EF, ToM) (see Figure 1). This may provide inroads for intervention in each aspect of this model.

## Existing intervention methods for improving social emotional functioning in adolescents and school-aged children with DLD

The difficulties described above indicate the need for interventions to improve social communication and cognitive skills in adolescents with DLD. Here, we will provide an overview of studies that tested the effectiveness of interventions targeting cognitive and social communication skills that underly social-emotional functioning in adolescents with DLD. Since we only found few studies that evaluated the effectiveness of interventions improving social emotional functioning in adolescents with DLD, we also reviewed some effect studies in school-aged children DLD. Computer searches of electronic databases (i.e., Web of Science, Google Scholar, PsycINFO) were conducted to locate appropriate studies. We reviewed effect studies that met the following selection criteria: (10 focusing on outcomes of social (communication) skills, pragmatic skills, social emotional functioning, EF, ToM, and SDS, (2) in school-aged children (>6 year) and adolescents with primary disabilities in the area of language (i.e., DLD, Specific Language Impairments [SLI], Pragmatic Language Impairments [PLI]), (3) were written in English, and (4) that had been published since 1985. In addition, we reviewed the references of selected studies. To determine the strength of evidence for efficacy or effectiveness we evaluated each study's control conditions, sample size and generalizability of findings (31).

### Social communication interventions

The important role of social communication indicates the need for interventions to improve language skills to use within social interaction (i.e., pragmatic language skills) (32). The study by (33) of one adolescent (15 year) with DLD showed improvements in social responsiveness, in comparison with one baseline measurement. Improvements were noted in both training and generalization contexts (i.e., based on parent observation). However, automation of these strategies resulting in a lasting effect was not achieved. Moreover, as there was only one participant and no baseline period or control group, it was unclear whether improvement was due to the intervention or to attention and stimulation. The study of Fujiki et al. (34) in four school-aged children (6–9 years), with language impairments (>1 SD below mean) analyzed the effectiveness of a social communication intervention. Within this intervention, children practiced and discussed specific ‘validating comments' (e.g., sharing information, asking peers questions etc.). Three of the four children showed increases of validating comments on pre tasks. Teachers rated overall likeability and prosocial behavior in two children. However, the small group sizes and lack of control group limited this findings. A study by Adams et al. (35) assessed an eight-week pragmatic intervention in six school-aged children (6–9 years) with PLI. The six children practiced different pragmatic and communication skills (i.e., practicing introductions, equal participation in talk). All six children showed improvements on some conversational task, which demonstrates the possibility to improve pragmatic skills by intervention. However, there was no control condition and generalization effects were not measured. A RCT by Adams et al. (36) in 57 school-aged children (5–10 years) with pragmatic language impairments (PLI) showed significant intervention effects on pragmatic functioning and social communication skills rated by parents. However, no significant changes were found for general language skills, in comparison with the control group. The study of Merrison (37) in nine (3 with PLI, 3 with SLI and 3 TD) school-aged children (7–11 years) analyzes the effectiveness of 6-weeks intervention at explicitly teaching pragmatic skills (i.e. communication repairs). All groups showed improvements in communication repairs, at post and follow-up measurements, supporting the idea that pragmatic skills can be explicitly taught. However, the small sample sizes, absence of control group, and real-life data limit the generalization of findings. A study by (38) tested the effect of a pragmatic skills intervention in one school-aged child (8 year) with DLD. The 12-weeks intervention focused on instruction (i.e., importance of conversations), modeling (i.e., video-models) and role-playing. The study found positive intervention effects on target verbal and non-verbal communicative skills, conversation topic maintenance and turn-taking skills, in comparison with the baseline. However, generalization was not directly assessed. In sum, pragmatic interventions show some preliminary evidence. However, the small sample sizes, heterogeneous diagnostic groups (i.e., DLD/PLI) and the absence of real-life data, limit the generalization of findings. Moreover, the only larger randomized study did not find effects on all primary outcomes.

### Theory of mind interventions

Emotion recognition skills and usage of underlying emotion words have been suggested to be important for the development of ToM (39, 40). Despite this importance, to our knowledge there are no studies evaluating the effects of emotion word learning and/or emotion recognition training in adolescents with DLD.

One study examining the effects of emotion word learning, in three school-aged children (5–10 years) with DLD, found improvements in the targeted skill (41). This 20-week emotion word learning program, improved the ability to use emotion words more adequately, in comparison with the baseline period. However, there were no measurements of generalization.

Training of sentential complements, an embedded preposition which can be false or true without affecting the truth value of the whole (i.e., “Lens said he saw a green tiger”), has also been suggested to be important for the development of ToM. Children have been trained in completing sentences, or to give answers on questions consisting of verbs of communication (i.e., say, ask, etc.) and/or mental state terms (i.e., think, know, etc.). To the best of our knowledge, there are no studies focusing on sentential complements in adolescents with DLD. A study examining the effects of training sentential complements in 13 school-aged children (6–12 years) with DLD found improvements in complements and ToM, in comparison with the controls (42). However, there were no measurements of generalization.

In sum, there is preliminary evidence for training emotion recognition skills, emotion words and sentential complements in school-aged children with DLD. However, the small number of studies, small sample sizes and the lack of real-life data limit the generalization of findings.

### Executive Functioning interventions

Studies examining the possible effects of EF training in children and adolescents with DLD are scarce. One study of a computer-based EF training in 10 school-aged children (8–12 years) with DLD, showed significant improvements at 6 months follow-up on the three trained EF tasks (43). During 25 sessions, children trained visuospatial working memory, inhibition and cognitive flexibility in a game-like environment. Besides significant results on the trained EF skills, there were no measurements of generalization to social skills or daily situations. In sum, since there is only one study with a small sample size, it is unclear whether results can be generalized.

### Self-directed speech intervention

The only self-directed speech training was performed at a slightly younger age. This self-directed speech training in 187 school-aged children (4–7 years) with DLD found improvements in self-directed speech, planning and problem solving, in comparisons with the control condition (44). This study showed that scaffolding can be effective for improving self-directed speech during planning and problem solving. However, lack of real life data and no measurements of other EF tasks, limit the generalization of findings.

## Discussion

The aim of this mini review was to provide an overview of studies examining the effectiveness of interventions targeting the factors underlying social-emotional functioning of adolescents with DLD. Based on this mini review, we conclude that little is known about the effectiveness of interventions targeting social-emotional functioning in adolescents with DLD. Studies examining the effectiveness of social communication or cognitive (EF, ToM, SDS) interventions in school-aged children and adolescents with DLD, showed preliminary evidence for improvements in the specific trained skill. Yet the strength of evidence for each intervention type is limited by modest study quality, as indicated by small sample sizes, frequent lack of control groups, and scarcity of real-life primary outcome measures. In addition, there were no studies measuring the effect of mixed interventions in children or adolescents with DLD. The studies reviewed were conducted in well-educated, industrialized, rich and democratic country's (i.e., America, England, France and the Netherlands). It is interesting to know if and how social emotional problems are trained in other countries.

To provide at least some recommendations for mixed interventions in adolescents with DLD, we reviewed some mixed interventions in adolescents with Autism Spectrum Disorder (ASD), as both adolescents with DLD and ASD have social-emotional problems (45). For example, the study of Bauminger (18) in 15 children and adolescents with ASD showed improvements in the ability to share experiences with peers, show interest in peers and sharing complex emotions. Teachers reported improvements in overall social skills, which is first evidence for generalization. Limitation of the study was the lack of a control group. The study of Gabbatore et al. (46) based on 21 adolescents with ASD showed intervention effects on linguistic (i.e., comprehension and production of communication acts), extralinguistic (i.e., gestures), paralinguistic (i.e., comprehension and expression of facial expressions and prosody) and context scales (adequacy of social norms), in a follow-up measurement. However, a control sample was not present. The study of Laugeson et al. (47) in 28 adolescents with ASD found improvement in social skills knowledge, social responsiveness and social skills as reported by parents and teachers, in comparison with the control group. In sum, mixed interventions in youth with ASD provide preliminary evidence on social emotional functioning. However, the small sample sizes and lack of control conditions, limits the generalization of findings.

A recurrent theme is the importance of practicing in real life. Yet realistic practice and scaffolding of social behaviors seems difficult to attain within practical and ethical boundaries. Fortunately, recent studies suggest potential benefits of using interactive Virtual Reality (VR) for improving social-emotional skills in youth with ASD (48, 49). Since VR interventions seems to improve social skills in adolescents with other (neuro)psychological problems, it may also be a promising technique for adolescents with DLD. First, real-life scenarios in VR may increase the generalization of the learned cognitive and social communicative skills. Second, the real-world environment in VR eliminates the need for the complex linguistic and mentalizing skills involved in traditional CBT, so that children with DLD may have more cognitive resources left at their disposal for social reflection and task attention (33). Third, recognizing emotions and responding to others' feelings and thoughts in real-life VR environments seem to improve the mentalizing and emotion recognition skills (49, 50). Fourth, VR may provide a safe and reliable environment: children can make mistakes without any chance of victimization, rejection or other risks that are associated with role playing in face-to-face interventions (48). Last but not least, therapeutic treatment *via* VR may increase the adolescents motivation, since most adolescents are fascinated by computers, videogames, and new techniques (48, 51).

Based on the theoretical framework and reviewed interventions we tentatively suggest potential key elements of interventions to promote social functioning by adolescents with DLD. First, we recommend interventions for improving social emotional functioning to teach linguistic, social communication (pragmatic) skills, and cognitive components (EF, ToM, SDS) underlying social emotional functioning. Second, it seems effective to give direct instruction of social communicative and pragmatic skills. The third proposed key element is analyzing social video-clips. Video-clips can help adolescents to recognize conversational cues in a specific real social situation with real peers (17). Fourth, for improving ToM it seems important to specifically teach emotion words, facial emotion recognition and sentential complements. Finally, it is suggested that role-playing in meaningful and real-life scenarios may be effective for the generalization of the trained social communicative skills.

There are a number of limitations concerning the present review of interventions for social emotional functioning. The first limitation is that (due to the lack of effect studies in adolescents) most of the reviewed studies have been conducted in school-aged children with DLD. Since adolescents with DLD are in a different stage of life, they may respond differently to the intervention method. The second limitation is that most reviewed studies included a small sample. Because of these small samples it is uncertain whether results can be generalized to the overall population. A third major limitation is that the reviewed studies lack primary outcomes to test generalization to daily life. As such, the outcomes of the studies support the feasibility of the intervention to improve social behavior or cognitive skills, but the generalizability of these results has not been demonstrated. The final limitation is that this mini review does not include studies focusing on more general language abilities (e.g., grammar, vocabulary). Thus, it is likely that a number of promising intervention practices with potential benefit for improving social emotional functioning were not reviewed.

Of course our findings depends on how we searched. In recent years, different terms have been used to label language problems in adolescents. Historically, the term SLI was used by many researchers, but recently the term DLD has been used more often. Therefore, we searched for effect studies in both SLI and DLD. In addition, we searched for studies focusing at PLI since we take a neuropsychological view at language problems. Searches that only focus on studies at DLD do not provide a complete overview of results.

In conclusion, interventions to improve social emotional functioning in adolescents with DLD should target the linguistic, social communication (pragmatic) skills, and cognitive components (EF, ToM, SDS) underlying social emotional functioning. In addition, interventions should provide opportunities to explicitly train these skills through social dialogue with others, to initiate internalization. Finally, we suggest to practice these skills in meaningful and real-life (VR) situations with peers.

## Author contributions

EA focused on, wrote the theoretical, empirical part of the mini-review, and integrated the empirical findings. CV, BO, EL, and BE finalized the mini review. All authors contributed to the article and approved the submitted version.
